# Neuroinflammation and Myelin Status in Alzheimer's Disease, Parkinson's Disease, and Normal Aging Brains: A Small Sample Study

**DOI:** 10.1155/2019/7975407

**Published:** 2019-07-04

**Authors:** Fei Han, Richard J. Perrin, Qing Wang, Yong Wang, Joel S. Perlmutter, John C. Morris, Tammie L. S. Benzinger, Jinbin Xu

**Affiliations:** ^1^Department of Radiology, Washington University School of Medicine, 510 S, Kingshighway Blvd, St. Louis, MO 63110, USA; ^2^Department of Pathology & Immunology, Washington University School of Medicine, 510 S, Kingshighway Blvd, St. Louis, MO 63110, USA; ^3^Knight Alzheimer Disease Research Center, Washington University School of Medicine, 510 S, Kingshighway Blvd, St. Louis, MO 63110, USA; ^4^Department of Neurology, Washington University School of Medicine, 510 S, Kingshighway Blvd, St. Louis, MO 63110, USA; ^5^Department of Neuroscience, Washington University School of Medicine, 510 S, Kingshighway Blvd, St. Louis, MO 63110, USA; ^6^Department of Physical Therapy, Washington University School of Medicine, 510 S, Kingshighway Blvd, St. Louis, MO 63110, USA; ^7^Department of Occupational Therapy, Washington University School of Medicine, 510 S, Kingshighway Blvd, St. Louis, MO 63110, USA

## Abstract

Microglia and astrocytes play important roles in mediating the immune processes and nutritional support in the central nervous system (CNS). Neuroinflammation has been indicated in the progression of neurodegenerative diseases Alzheimer's disease (AD) and Parkinson's disease (PD). Chronic neuroinflammation with sustained activation of microglia and astrocytes may affect white matter tracts and disrupt communication between neurons. Recent studies indicate astrogliosis may inhibit remyelination in demyelinating disorders such as multiple sclerosis. In this study, we investigated the relationship between neuroinflammation and myelin status in postmortem human brain tissue (*n* = 15 including 6 AD, 5 PD, and 4 age-matched, neurologically normal controls (NC)). We conducted systematic and quantitative immunohistochemistry for glial fibrillary acidic protein (GFAP), ionized calcium-binding adaptor molecule 1 (Iba1), amyloid beta, and highly phosphorylated tau (tauopathy). White matter intactness was evaluated by myelin and axon staining in adjacent brain tissue sections. Eight of 15 cases (4 AD, 3 PD, and 1 NC) showed increased immunoreactivity for microglia and astrocytes in the white matter that connects striatum and cortex. Quantitative analysis of these 8 cases showed a significant negative correlation between GFAP (but not Iba-1) and myelin (but not axon) staining in white matter (*r*^2^ = 0.78, *p* < 0.005). Tau, but not amyloid beta plaques, is significantly higher in AD vs. PD and NC. Tau burden increases with age in AD cases. These observations indicate that astrocytosis in white matter is associated with loss of myelin in AD, PD, and normal aging and that tau is a potent biomarker for AD.

## 1. Introduction

White matter disease is a common pathology involved in the dementia of Alzheimer's disease [1, 2], multiple sclerosis [3], and cerebrovascular disease [4]. Brain white matter is generally categorized into two types: deep and superficial white matter. Compared with deep white matter, superficial white matter undergoes later myelination and has less wraps around the axon. These features render it vulnerable to aging- or injury-related impairment [5]. Moreover, superficial white matter has complex connectivity with cortical fibers and contributes to information processing and integration [6]. This disorder of superficial white matter has been implicated in cognitive dysfunction, such as Alzheimer's disease, schizophrenia, and Huntington's disease [7]. Astrocytes are the most numerous cell type in the CNS and perform many physiological functions in this system. These functions include maintaining homeostasis at the synapse, regulation of neuronal signaling, protecting neurons from oxidative damage, and assisting in the formation of myelin. Astrocytes not only control proliferation and migration of oligodendrocytes but also regulate the timing of myelination [8]. Some studies suggest that GFAP knockout mice have more extensive dysfunction in the white matter than in the gray matter, indicating that astrocytes play crucial roles in facilitating and maintaining normal myelination [9]. However, overactivated astrocytes also secrete factors associated with the inhibition of myelination, such as transforming growth factor (TGF-*α*) and bone morphogenetic protein (BMP) 2/4 [10].

In addition, cerebral amyloid-beta (A*β*), neurofibrillary tangles (abnormal tau), and neuroinflammation are all involved in AD pathology [11]. Dysfunctional perivascular cells also play a pathological role in neurodegenerative diseases and cerebral vascular diseases [12]. In the current study, we examined 15 autopsied human brains that included 5 cases of Parkinson disease (PD), 6 cases of Alzheimer disease (AD), and 4 cases of age-matched cognitively normal controls (NC) using immunohistochemistry (IHC) staining for GFAP and Iba1, A*β* and phosphorylated tau, and *α*-SMA, as well as myelin and axon staining. We found that cases with high GFAP expression (4 AD, 3 PD, and 1 NC) in superficial white matter have a significantly negative correlation with myelin loss, whereas cases with low GFAP expression (2AD, 2 PD, and 3 NC) do not present negative correlation. Phosphorylated tau was highly expressed and increased with age in AD, and tau burden was significantly higher than A*β* in AD cases. These data suggest that chronic inflammation-induced astrocytosis in superficial white matter might be associated with myelin impairment and disease progression in these patients. Furthermore, it suggests that tau is a more potent biomarker than A*β* for aging-associated dementia in AD.

## 2. Materials and Methods

### 2.1. Cases

Postmortem human brain tissue samples including six AD, five PD, and four age-matched cognitively healthy controls (3 female and 12 male) were obtained from the Charles F. and Joanne Knight Alzheimer's Disease Research Center (AD and control cases) and the Movement Disease Research Center (PD cases), Department of Neurology, Washington University School of Medicine (Table 1). Written consent from the participant or the next-of-kin was obtained for brain removal, following local Institutional Review Board policies and procedures at Washington University in St. Louis.

### 2.2. Tissue Collection, Staining, and Immunohistochemistry

Briefly, brains were removed at the time of autopsy and the left hemispheres were fixed in 10% neutral-buffered formalin for 2 weeks or more before samples were processed for histology and embedded in paraffin. Histologic sections were cut at 6 *μ*m thickness and mounted on glass slides. Histologic stains included hematoxylin and eosin, Luxol fast blue and Cresyl echt violet (NeuronMyelinStain), and Bielschowsky silver staining (Hitobiotec Corp. Kingsport, TN, USA). For immunohistochemistry, all specimens underwent heat-induced antigen retrieval (0.01 M citrate buffer at pH 6.0) and were immunostained by the EnVision method. Specimens were then incubated overnight at 4°C with rabbit monoclonal anti-GFAP antibody (1 : 250 dilution, Abcam Inc. MA, USA), rabbit polyclonal anti-Iba1 antibody (1 : 250 dilution, Wako Chemicals USA, Inc), and rabbit polyclonal to alpha smooth muscle actin antibody (1 : 100 dilution, Abcam Inc. MA, USA). Avidin-biotin complex (VECTASTAIN® UNIVERSAL ELITE ABC KIT) was used for immunohistochemical staining of A*β* (10D5, Elan Pharmaceuticals, San Francisco, CA, USA) and phosphorylated tau (PHF-1, generous gift of Dr. Peter Davies, Feinstein Institute for Medical Research, Manhasset, NY, USA), phosphorylated TDP-43 (Cosmo Bio USA Inc, Carlsbad, CA, USA), phosphorylated *α*-synuclein (Wako Chemicals USA Inc, Richmond, VA, USA), and PBS without the primary antibody for a negative control. Specimens were then incubated with horseradish peroxidase-linked goat anti-rabbit or mouse secondary antibody and ABC complex, followed by reaction with diaminobenzidine. No specific immunostaining was detected in negative controls under these conditions. The positive signaling for GFAP and Iba1 was located in the cytoplasm, and their positive areas and densities of myelin and axon were analyzed by VISIOPHARM imaging analytic software. The severity of Lewy body pathology was assessed using the Braak et al.'s Parkinson's disease staging scale [13]. Alzheimer's disease neuropathological changes were assessed by the Braak staging method [14, 15].

### 2.3. Statistical Analysis

All values are expressed as mean ± SEM. Mann–Whitney nonparametric test and one-way analysis of variance (ANOVA) were used to identify significant differences in two or multiple comparisons. Pearson correlation coefficients and two-tailed hypothesis test were used to evaluate correlation between two variables. All analyses were performed by GraphPad Prism software. A level of *p* < 0.05 was considered statistically significant.

## 3. Results

### 3.1. Clinical Features of 15 Cases

As shown in Table 1, there are 6 cases of AD, 5 cases of PD, and 4 cases of age-matched neurologically normal controls, with the median age of 78 (69–93) years including 3 females and 12 males. There are no significant difference in the average age at death (*p* > 0.8, Student's *t*-test), postmortem interval (*p* > 0.19, Student's*t*-test), or brain weight (*p* > 0.28, Student's *t*-test) among PD, AD, and NC. Only one patient with PD had bilateral subthalamic nucleus-deep brain stimulation.

**Figure 1:**
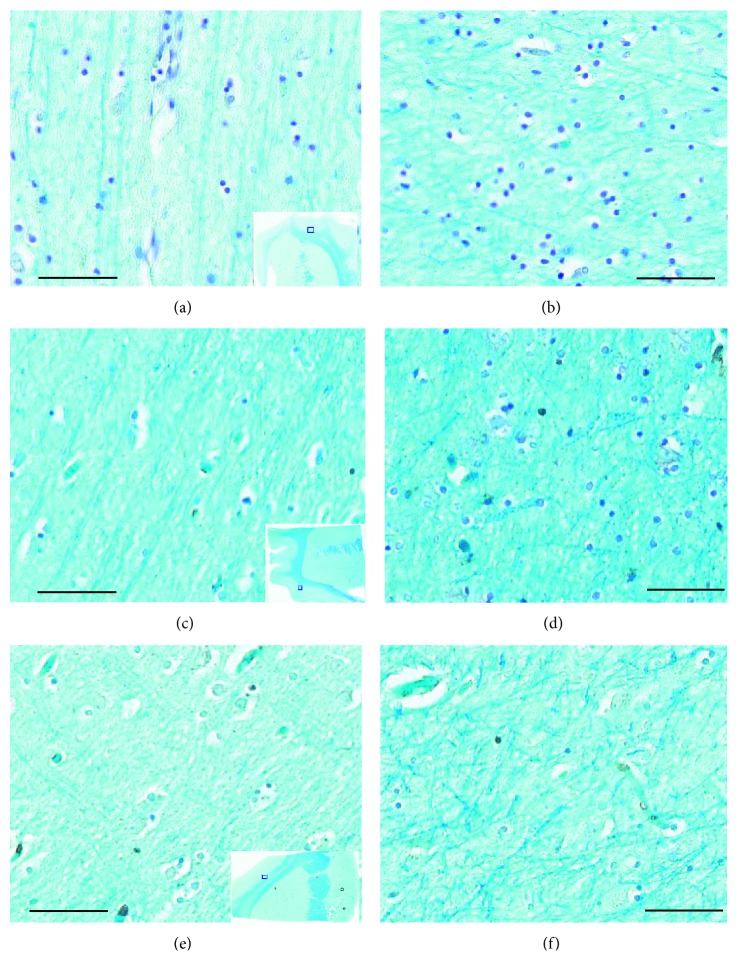
Myelin staining in the representative AD, PD, and age-matched cognitive intact controls (NC) cases. (a, c, e) The connected fine fibers between the junction of subcortical white and gray matter from AD, PD, and NC, respectively (the bars represent a 200 *μ*m scale; the insets show a gross view, and the small squares indicate the zoomed regions). (b, d, f) The fibers in the subcortical white matter from the same AD, PD, and NC cases, respectively. The fibers are short and small in diameter, they are arranged in complex cross-linking pattern (the bars represent a 200 *μ*m scale).

All the white matter regions originating from these samples were identified as superficial white matter by histological observation (Figure 1).

### 3.2. Expression of GFAP and Iba1 in the Astrocytosis Group and Nonastrocytosis Group

**Figure 2:**
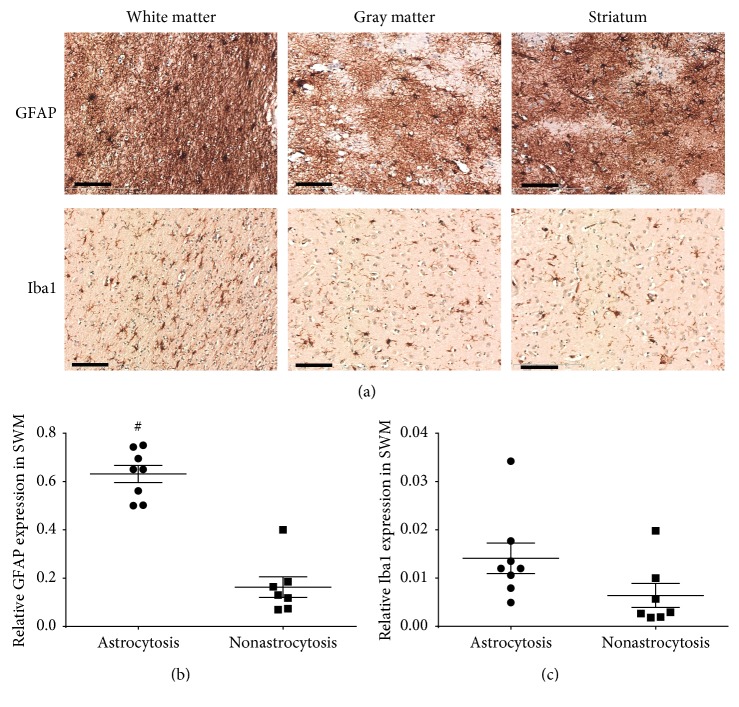
Astrocytes and microglia in the striatal tissue sections using GFAP and Iba1 antibodies. (a) Representative images of activated astrocytes and microglia in white matter, gray matter, and striatum. The activated astrocytes showed enlarged cell bodies and enlongated fibers, while activated microglia showed meatball-like appearance with enlarged cell bodies and shortened processes. The scale bar represents 100 *μ*m. (b, c) The quantitative analysis of the GFAP and Iba1 expression in white matter in the astrocytosis and the nonastrocytosis groups. The bars represent mean ± SEM. ^*∗*^represents *p* < 0.05.

The expression of GFAP and Iba1 was detected by immunohistochemistry staining. The activated astrocytes and microglia are shown in Figure 2(a). The quantitative expression of GFAP and Iba1, shown in Figures 2(b) and 2(c), revealed significantly higher expression of GFAP (but not Iba1) in the superficial white matter of the astrocytosis group compared to the nonastrocytosis group (^*∗*^*p* < 0.05).

### 3.3. Different Functional States of Microglia Were Observed in AD, PD, and NC Samples

**Figure 3:**
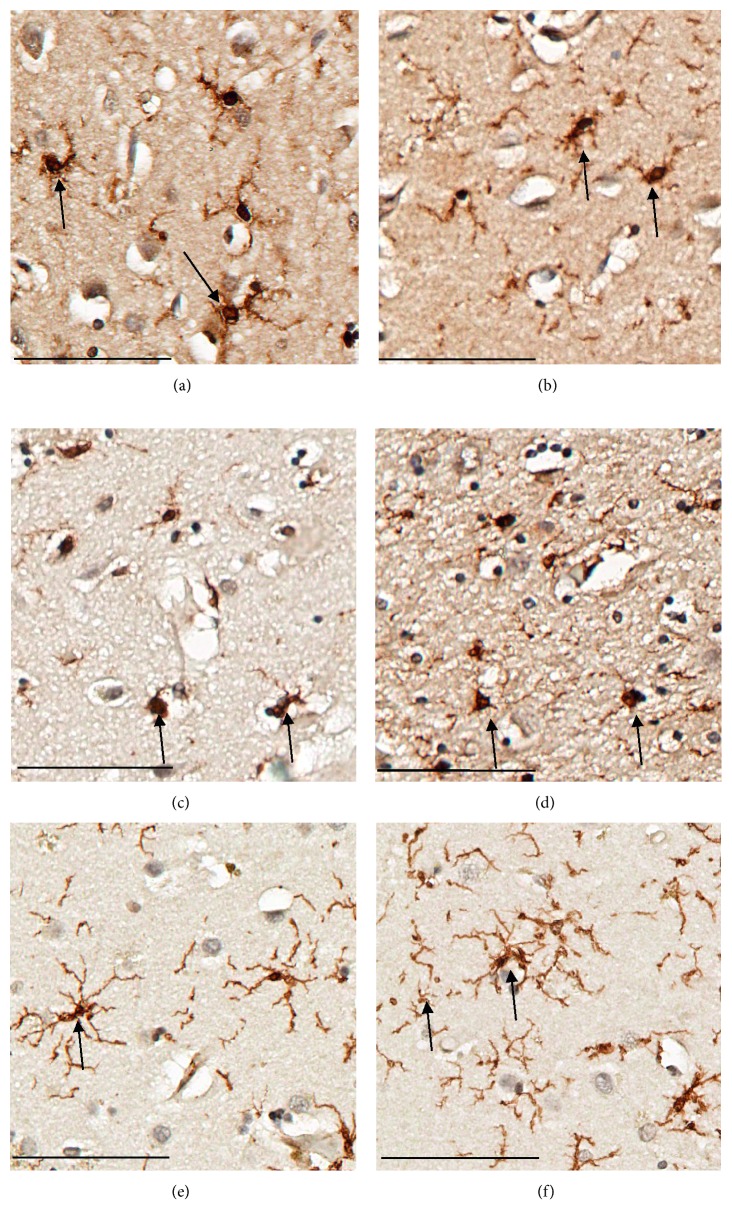
Different morphologies of microglia in AD, PD, and NC. (a, b) Dystrophic microglia from striatal white matter and cortex of two AD patients which displayed twisted or fragmented processes and cell body enlargement. (c, d) Activated and dystrophic microglia from striatum of two PD patients which showed cell body enlargement and fragmented processes. (e, f) Resting microglia from striatum of two health controls which showed the small cell body with highly branched processes. The scale represents 400 *μ*m.

Compared to cognitive intact healthy control, more dystrophic and activated microglia were spotted in AD and PD patients. These findings suggest not only the activation but also the underlying degenerative process of microglia contribute to pathologenesis of AD and PD (Figure 3).

### 3.4. Negative Correlation between the GFAP Expression and Myelin Density in the Superficial White Matter of Astrocytosis Group (4 AD, 3 PD, and 1 NC)

**Figure 4:**
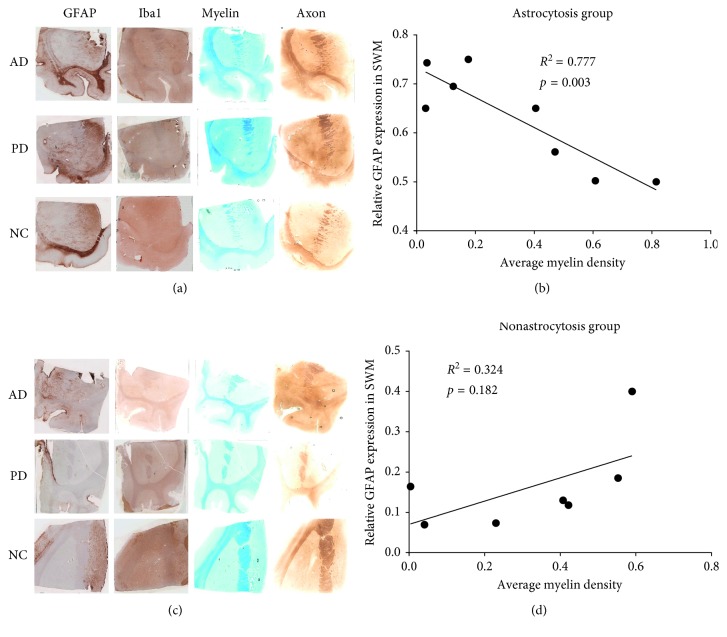
GFAP, Iba1, myelin, and axon staining and correlation analysis between GFAP expression and myelin density. Left panel shows the representative IHC images of GFAP, Iba1, myelin, and axon staining in the striatal tissue sections from AD, PD, and NC cases in the astrocytosis (a) and the nonastrocytosis group (c). Right panel shows the correlation between GFAP expression and myelin density in the astrocytosis (b) and the nonastrocytosis (d) groups.

**Figure 5:**
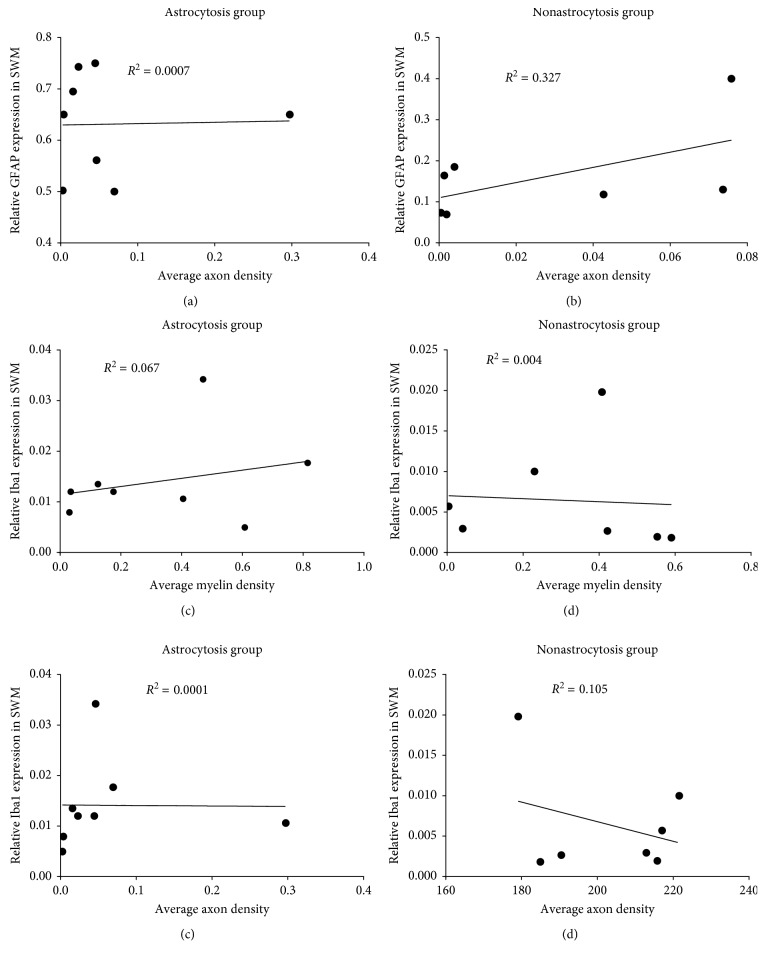
Correlation analysis between GFAP and Iba1 staining with axon and myelin densities. (a, b) The correlation between the expression of GFAP and axon densities in the astrocytosis and nonastrocytosis group. (c, d) The correlation between the expression of Iba1 and myelin densities in the astrocytosis and nonastrocytosis group. (e, f) The correlation between the expression of Iba1 and axon densities in the astrocytosis and nonastrocytosis group.

After classifying these cases into two groups based on expression of GFAP in superficial white matter, the astrocytosis group which has high GFAP expression and includes 4 AD, 3 PD, and 1 NC cases and the nonastrocytosis group which includes 2 PD, 2 AD, and 3 NC cases. Representative images of AD, PD, and NC patients for GFAP, Iba1, and myelin and axonal staining are shown in Figures 4(a) and 4(c). As shown in Figures 4(b) and 4(d), the expression of GFAP significantly inversely correlates with myelin densities of superficial white matter in the astrocytosis group (*R*^2^ = 0.78, *p*=0.003), but GFAP expression does not correlate with myelin densities of superficial white matter in the nonastrocytosis group (*R*^2^ = 0.32, *p*=0.18). However, as shown in Figure 5(a), the expression of GFAP does not significantly correlate with axon density in the astrocytosis group (*R*^2^ = 0.0007, *p*=0.35) nor in the nonastrocytosis group (*R*^2^ = 0.33, *p*=0.18), which suggest that astrocyte may also contribute axon generation in white matter. Although a high but not significant expression of Iba1 was detected in the astrocytosis group compared to the nonastrocytosis group, the expression status of Iba1 does not correlate with myelin or axon densities whether in the astrocytosis group or the nonastrocytosis group (Figures 5(c)–5(f)).

The expression of GFAP and Iba1 in different regions of 6 cases of AD, 5 cases of PD, and 4 cases of age-matched cognitively intact controls.

**Figure 6:**
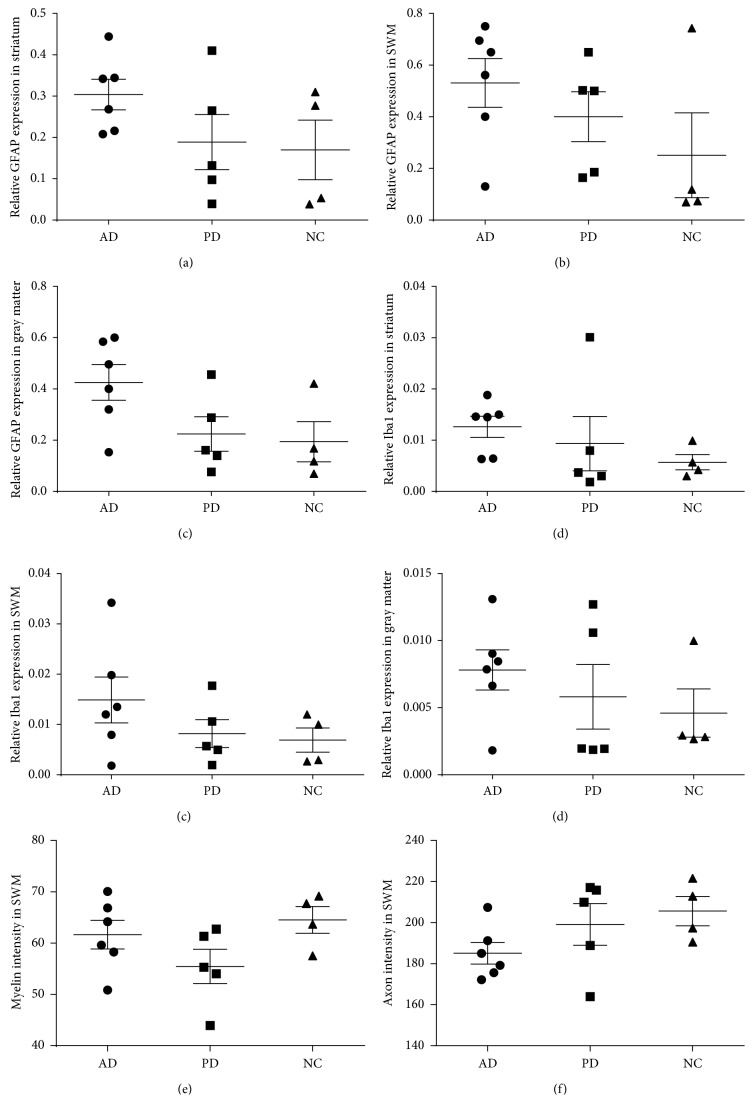
Relative GFAP and Iba1 expression and myelin and axon status in PD, AD, and age-matched cognitive intact controls (NC). (a) Expression of GFAP in the striatum. (b) Expression of GFAP in white matter. (c) Expression of GFAP in gray matter. (d) Expression of Iba1 in the striatum. (e) Expression of Iba1 in white matter. (f) Expression of Iba1 in gray matter. (g) Myelin status in AD, PD, and NC. (h) Axon status in AD, PD, and NC. The bars represent mean ± SEM.

We also analyzed the expression of GFAP and Iba1 in superficial white matter, cortical gray matter, and striatum in these patients (6 AD, 5 PD, and 4 NC). As shown in Figures 6(a)–6(h), there is no significant differences in expression of GFAP and Iba1 in AD, PD, and NC groups in different regions (*p* > 0.05), probably due to small sample limitation. Furthermore, we analyzed myelin and axon status in superficial white matter, there is no significant differences except mild decline of myelin sheath observed in PD patients.

### 3.5. Tauopathy and Pericyte Status in 6 Cases of AD, 5 Cases of PD, and 4 Cases of Age-Matched Cognitively Intact Controls

**Figure 7:**
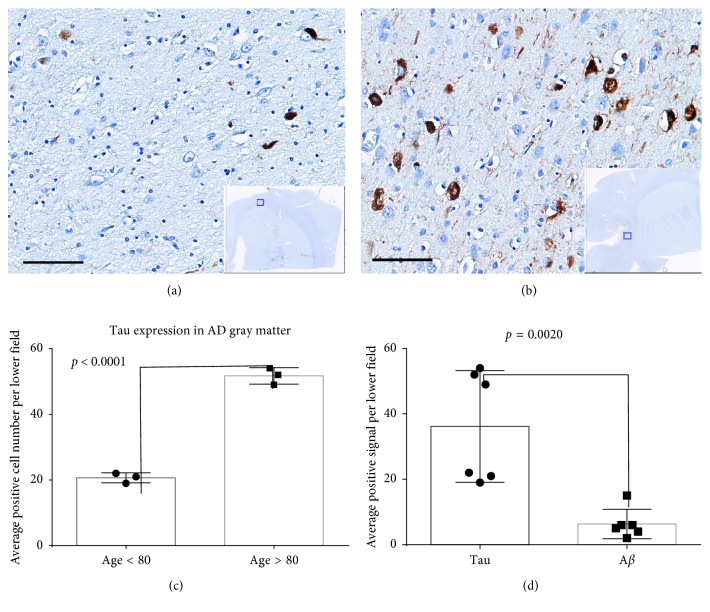
Abnormal tau expression in AD patients. (a) Neurofibrillary tangle deposition in a 75-year-old male patient. (b) Neurofibrillary tangle deposition in a 93-year-old male patient (insets represent gross image, the small squares indicate the zoomed regions, and the bars represent a 200 *μ*m scale). (c) Quantitative analysis of tau expression in six AD cases. (d) Quantitative analysis of tau and A*β* expression in the gray matter of six AD cases.

**Figure 8:**
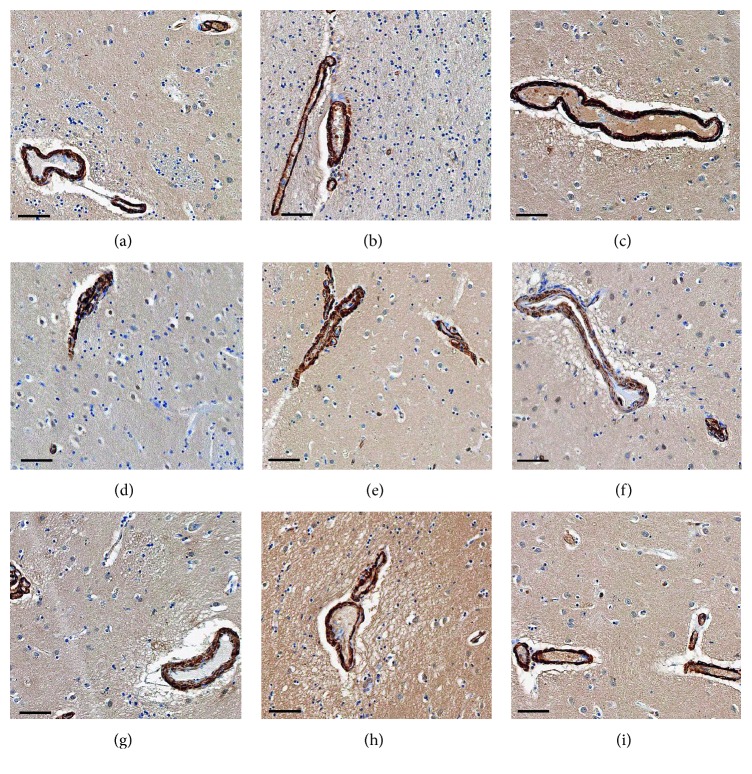
Immunohistochemical staining of *α*-SMA in AD, PD, and age-matched cognitively normal controls (NC) cases. The positive staining (brown color) were localized in the perivascular area (pericytes). The nucleus was stained by hematoxylin. The bars represent a 100 *μ*m scale. (a) AD1, (b) AD2, (c) AD3, (d) PD1, (e) PD2, (f) PD3, (g) NC1, (h) NC2, (i) NC3.

Hyperphosphorylated tau was identified by labeling with a PHF mouse monoclonal antibody, and tau was found to be extensively expressed in all six AD cases. Tau was not, or rarely, expressed in PD and NC cases. The positive areas of tau in AD cases were primarily localized in the gray matter and junction between gray and white matter. Intracellular tau expression was found to be localized in the cytoplasm of neurons and gliocytes. Phosphorylated tau was highly expressed in AD cases. We classified these cases into two age groups (under 80 and over 80). Tau expression was observed to be significantly higher in the >80 group than in the <80 group (Figure 7). Amyloid beta (A*β*) was labeled by a 10D5 antibody, and A*β* plaque burden was found to be significantly lower than tau in these AD cases (Figure 7). Dysfunction of perivascular cells was also reported to be involved in the neuropathological diseases, such as Alzheimer's disease and multiple sclerosis. Here, we investigated pericytes status by labeling *α*-SMA, a specific marker for pericytes. No perivascular cytopathology was identified in these cases (Figure 8). However, we cannot exclude the presence of perivascular cytopathology in other brain regions due to our sample limitations.

## 4. Discussion

Chronic neuroinflammation, mediated by reactive astrocytes and activated microglia, plays an important role in the progression of neurodegenerative diseases, such as Parkinson's disease and Alzheimer's disease. In this study, we assessed neuroinflammation in the striatum and associated cortical gray matter and white matter from 15 postmortem human brains including 6 cases of AD, 5 cases of PD, and 4 neurologically intact age-matched controls. Although our data suggest that there is a trend that AD cases (with high burden of beta amyloid and tau) have more neuroinflammation than PD and control cases, we found no significant differences for activation and proliferation of astrocytes and microglia either in cortical gray matter, superficial white matter, or striatum among PD, AD, and controls. This is consistent with translocator protein binding (TSPO) or previous peripheral benzodiazepine receptor (PBR) quantitative autoradiography studies using [^3^H]PK11195 and [^3^H]PBR28 in postmortem frozen human brains; TSPO binding density was found to be not significantly different among AD (*n* = 7), dementia with Lewy bodies (DLB) disease (*n* = 5), or cognitively intact age-matched controls (*n* = 8) in these brain regions [16]. However, some cases in the current study (4 AD, 3 PD, and 1 NC) showed a significantly higher expression of GFAP in the superficial white matter (>0.5 positive staining area), in comparison to the other cases (2 AD, 2 PD, and 3 NC) (<0.4 positive staining area). Based on this difference, we classified these cases into two groups: astrocytosis (4 AD, 3 PD, and 1 NC) and nonastrocytosis (2 AD, 2 PD, and 3 NC). Staining of myelin and axon was performed to investigate the relationships between the activation of glia cells (astrocyte and microglia) and neurodegeneration, as reflected by axonal and myelin integrity in the subcortical-striatal superficial white matter. We found that GFAP expression significantly negatively correlated with myelin density in superficial white matter in the astrocytosis group, but not in the nonastrocytosis group. In contrast, Iba1 staining did not correlate significantly with myelin density in either group.

Myelin sheath can boost neural transduction via the node of Ranvier by insulating nerve cell axons in the central and peripheral nervous systems. Damaged myelin sheath could be a biomarker for the early-stage axon degeneration. It has been reported that demyelination could induce axon abnormalities by reducing axon caliber, abnormal neurofilament distribution, and increasing mitochondria number [17–19]. However, a study posited that axonal degeneration could occur independently from myelin loss [20]. Here, we also investigated axon density in superficial white matter and evaluated its association with the expression of GFAP or Iba1 and myelin densities in superficial white matter. Myelin density did not significantly correlate with axon density in all 15 cases (data not shown), nor did axon density significantly correlate with GFAP or Iba1 expression.

Tau pathology has been a hallmark of Alzheimer's diseases (AD). Tau exists as monomers, paired helical fiaments (PHFs), and straight filaments in AD [21]. Here, we investigated tau status by labeling with a PHF antibody in the striatal sections of AD, PD, and NC cases. Phosphorylated tau was highly expressed in all AD patients, especially in the age >80 group, compared to PD and NC cases. Furthermore, we also conducted amyloid beta immunohistochemistry in the adjacent sections of all cases. No concurrent expression of phosphorylated tau and amyloid beta was found in these cases except for one case, 83-year-old female. This case had a decent expression of amyloid beta in the gray matter area. The overall expression of amyloid beta was significantly lower compared to phosphorylated tau in all AD cases. No obvious expression of amyloid beta was observed in all PD and NC cases. These data support the hypothesis that tau pathology is independent of amyloid beta in the neurodegenerative diseases and that neurofibrillary tangles (tauopathy) are a better biomarker for predicting cognitive impairment in Alzheimer's diseases.

Pericytes, the perivascular-specialized smooth muscle cells, have multiple pathological functions in the brain. Interactions between pericytes and endothelial cells are important for the remodeling and maintenance of the vascular system. Pericytes also regulate the neurotransmitter transport and vascular permeability. Dysfunction of pericytes is involved in neuropathological diseases, such as hypertension, diabetic retinopathy, and Alzheimer's diseases [12]. Here, in this study, we also evaluated pericyte status by *α*-SMA staining. No obvious perivascular cytopathology was observed in the cases we studied.

Patients with neurodegeneration disease often have damage in the white matter, which is composed of axonal fibers interconnecting neurons in the brain [22]. White matter damage, which includes demyelination and inflammation, is associated with cognitive dysfunction and is one of the most important factors in the complex etiology of AD [23, 24]. Our study suggests that chronic activation of astrocytes and microglia may contribute to myelin sheath loss and nerve fiber degeneration. Abnormal tau was highly expressed in AD compared to PD and NC. Neurofibrillary tangles deposition is independent of plaques formation in the course of AD. No dysfunctional pericytes were observed in AD, PD and NC cases. Taken together, reactive astrocytes may produce toxic effects on myelin sheath and abnormal tau has a better correlation with AD dementia than amyloid beta.

## Figures and Tables

**Table 1:** Demographic and neuropathological features of subjects with PD or AD and age-matched controls.

Age (year)	Gender	Onset (years)	STN-DBS (years)	Clinical cause of death	Brain weight (g)	PMI (h)	Braak PD	Braak A*β*	Braak NFT	Diagnosis
84	F	67	0	Inanition	1190	8	5	0	2	PD
87	M	72	0	Inanition	1300	7.7	5	1	1	PD
73	M	58	0	Unknown	1500	52	5	1	2	PD
86	M	63	0	Inanition	1270	15	4	1	2	PD
76	M	66	75	Myocardial infarction	1400	40.5	5	0	1	PD
83	F	69	0	Inanition	900	10.5	0	5	5	AD
93	M	81	0	Inanition	1450	16	0	5	6	AD
85	M	75	0	Inanition	1130	16.5	0	5	5	AD
76	M	67	0	Inanition	1384	28	0	4	6	AD
75	M	67	0	Myocardial infarction	1430	6.5	0	3	3	AD
69	M	60	0	Non-Hodgkin's lymphoma	1350	21	0	4	6	AD
78	M	N/A	0	Pneumonia	1220	14	0	0	1	NC
78	M	N/A	0	Metastatic prostate cancer	1208	87	0	0	1	NC
78	F	N/A	0	Duodenal cancer	1290	15.7	0	0	2	NC
91	M	N/A	0	Septicemia	1250	16	0	0	2	NC

STN-DBS, subthalamic nucleus-deep brain stimulation; PMI, postmortem interval; Braak PD, Braak Lewy body stages in Parkinson's disease (range: 1–6); Braak A*β*, Braak beta-amyloid plaque stage; Braak NFT, Braak neurofibrillary tangle stage; PD, Parkinson's disease; AD, Alzheimer's disease; NC, age-matched cognitively intact normal control.

## Data Availability

The data we used are from quantification analysis from VISIOPHARM imaging analytic software. All these data used to support the findings of this study are included within the article and can be shared to anyone.
